# Panchromatic Image Super-Resolution Via Self Attention-Augmented Wasserstein Generative Adversarial Network

**DOI:** 10.3390/s21062158

**Published:** 2021-03-19

**Authors:** Juan Du, Kuanhong Cheng, Yue Yu, Dabao Wang, Huixin Zhou

**Affiliations:** 1 Xidian School of Physics and Optoelectronic Engineering, Xidian University, Xi’an 710071, China; jd_juandu@163.com (J.D.); hxzhou@mail.xidian.edu.cn (H.Z.); 2 School of Computer Science and Engineering, Xi’an University of Technology, Xi’an 710048, China; kh_cheng@163.com; 3 Beijing Institute of Spacecraft System Engineering, Beijing 100094, China; cast_wdb@126.com

**Keywords:** super resolution, attention-augmented convolution, panchromatic images, WGAN

## Abstract

Panchromatic (PAN) images contain abundant spatial information that is useful for earth observation, but always suffer from low-resolution ( LR) due to the sensor limitation and large-scale view field. The current super-resolution (SR) methods based on traditional attention mechanism have shown remarkable advantages but remain imperfect to reconstruct the edge details of SR images. To address this problem, an improved SR model which involves the self-attention augmented Wasserstein generative adversarial network ( SAA-WGAN) is designed to dig out the reference information among multiple features for detail enhancement. We use an encoder-decoder network followed by a fully convolutional network (FCN) as the backbone to extract multi-scale features and reconstruct the High-resolution (HR) results. To exploit the relevance between multi-layer feature maps, we first integrate a convolutional block attention module (CBAM) into each skip-connection of the encoder-decoder subnet, generating weighted maps to enhance both channel-wise and spatial-wise feature representation automatically. Besides, considering that the HR results and LR inputs are highly similar in structure, yet cannot be fully reflected in traditional attention mechanism, we, therefore, designed a self augmented attention (SAA) module, where the attention weights are produced dynamically via a similarity function between hidden features; this design allows the network to flexibly adjust the fraction relevance among multi-layer features and keep the long-range inter information, which is helpful to preserve details. In addition, the pixel-wise loss is combined with perceptual and gradient loss to achieve comprehensive supervision. Experiments on benchmark datasets demonstrate that the proposed method outperforms other SR methods in terms of both objective evaluation and visual effect.

## 1. Introduction

Panchromatic (PAN) images have been widely used in various applications, such as weather forecasts, environmental monitor, and earth observation. However, since the PAN images are always taken from space satellites with a large field of view, their spatial resolution is usually quite limited, and details of ground objects, for this reason, cannot be well distinguished. To resolve this problem, recent works began to focus on the super-resolution (SR) of PAN images. Due to the limitation of sensors, the PAN images captured from satellite sensors suffers from the heavy image degradation, which is an urgent need for SR to improve resolution and rich image texture through image processing algorithms.

The performance of SR algorithms [1,2,3,4] has been greatly boosted by the convolutional neural networks. The conventional supervised learning model tries to minimize the error between ground truth and SR results, whereas this design cannot well utilize the difference between those two samples semantically, arising from the fact that the loss functions are usually assigned as basic error functions, such as Mean Square Error (MSE), Structural Similarity Index Measurement(SSIM), or L1 Norm [5]. The the adversarial generative network (GAN) is introduced to resolve this shortage. Unlike normal generative networks, GAN-based methods apply the discriminative network to minimize the semantic distance between generated images and ground truth images through the discriminative error, producing High-resolution (HR) results with more details and naturalness. Although GAN-based SR models have made successful progress, there are still some limitations, such as the training instability and the limited representative ability of spatial-wise and channel-wise features [6]. The training instability is usually caused by the nonlinearity of the discriminative supervision, which may cause mode collapse. In addition, traditional convolution cannot respond to the different contribution among multi-channel and different locations of the feature maps.

Some improved models have been proposed to address the issue of feature representation in SR networks. Residual channel attention networks (RCAN) [7] is introduced to learn features across channels and enhance long-term information. Channel attention is used to exploit the features across different channels, but this design cannot fully use the the relevance among different locations of the feature. To further dig out the hidden relation within features, a channel and spatial attention block (CBAM) [8] is developed via combining channel-wise and spatial attention mechanisms into the network. Y.T. Hu first introduced the CBAM block into SR network [9], which integrates the CBAM features of the channel-wise attention and spatial attention into the residual block (CSAR) to modulate the residual features. The CSAR blocks are stacked in a chain structure to dynamically modulate multi-level features in a global-and-local manner. The multi-level features, in this way, can be adapted and fused with a hierarchical feature map through gated fusion. But, in fact, the relevant information between the channel feature and the spatial feature has not been excavated in CSAR blocks.

To effectively settle the above problem, attention-augmented convolution [10] is introduced in this paper to utilize the relevance among multiple features. Attention-augmented convolution improves classic convolution by augmenting the features and giving adaptive weight for feature combination, which can flexibly adjust the fraction of attentional channels to keep inter information among features. This allows the network to capture long-range interactions without increasing the number of parameters, whereas the self-attention mechanism has not been fully explored in SR.

In this work, a self augmented attention Wasserstein generative adversarial network (SAA-WGAN) is proposed for PAN images SR. We first integrate a convolutional block attention module (CBAM) into each skip-connection of the encoder-decoder subnet instead of stacking in a chain for CBAM features extraction in multi-scale. To obtain relevant information for hierarchical features, the self augmented attention (SAA) block using attention-augmented convolution are presented for extraction of the hinder feature and contextual information. In our SAA-WGAN, an encode-decode structure with CBAMs is used as one branch network, and the SAA block is utilized as another parallel branch, providing more helpful features in multiply scales and layers for the reconstruction of HR result. In addition, the pixel-wise information and high-level semantic information can be exploited by the combined loss of pixel loss and perceptual loss. As result, our method obtains better visual quality and recovers more image details compared with other state-of-the-art SR methods.

In summary, the main contributions of this paper are listed as follows:

(1) We propose a WGAN-based network (SAA-WGAN) for PAN image SR, which is integrated with the encode-decode structure and CBAM.
(2) We apply the self-attention module into the WGAN network, from which the long-range features can be well preserved and transferred.
(3) The generate loss is a combination of pixel loss, perceptual loss, and gradient loss to achieve the supervise in terms of both image quality and visual effect.
(4) Extensive evaluations have been conducted to verify the above contributions.

The remainder of this paper is organized as follows. We introduce the related Generative Adversarial Networks and Attention Features in Section 2. The proposed method of SAA-WGAN for PAN image super resolution is described in Section 3. The experimental results and analysis are reported in Section 4. The conclusion of this paper is stated in Section 5.

## 2. Related Work

### 2.1. Generative Adversarial Networks

**Figure 1:**
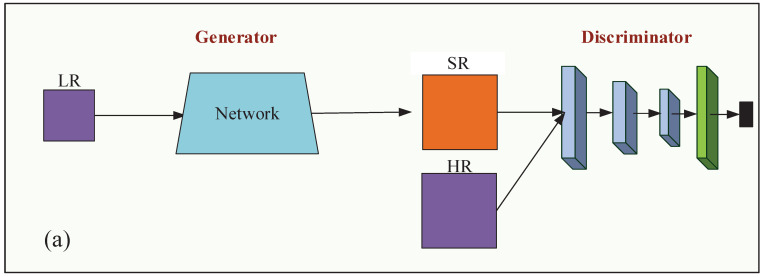
Structure of standard WGAN.

Traditional GANs [11,12,13] always narrow the gap between the generated sample and the real image by minimizing the Kullback–Leibler divergence (KL)distance between discriminative results, and the structure of gan is shown in Figure 1. The discriminator network in GAN can distinguish real and false samples, as well as produce very realistic SR results. Since the KL divergence is not linear for the input distribution space, which means the supervision of the discriminator is non-uniform for all the input samples, thus, the performance of traditional GANs is quite limited.

In SR, the generator network is trained to capture the real data distribution so that its generative samples can be as real as possible, which means to minimize $E\left[log\left(D\left(I,x\right)\right)\right]+E\left[log\left(1-D\left(I,\hat{x}\right)\right)\right]$. The discriminative network estimates the probability of a given sample coming from the real dataset, i.e., it can maximize the probability to distinguish SR sample from real data. So, the contest between the discriminator and the generator is usually formulated as a zero sum with cross-entropy targets.

(1) $$\begin{array}{c} \underset{G}{min}\underset{D}{max}E\left[log\left(D\left(I,x\right)\right)\right]+E\left[log\left(1-D\left(I,\hat{x}\right)\right)\right], \end{array}$$

where *x* is the input, $\hat{x}$ is the SR image, *I* is groundtruth, *D* is discriminator in the network, and *G* is generator in the network. Hence, the discriminator loss is

(2) $$\begin{array}{c} \underset{D}{Loss}=-E\left[log\left(D\left(I,x\right)\right)\right]-E\left[log\left(1-D\left(I,\hat{x}\right)\right)\right]. \end{array}$$

In practice, a modified generator loss is used:

(3) $$\begin{array}{c} \underset{G}{Loss}=-E\left[log\left(D\left(I,\hat{x}\right)\right)\right]. \end{array}$$

### 2.2. Attention Features

Attention has enjoyed widespread adoption in convolutional neural networks (CNN ) models, including SR networks, because of its ability to enhance feature representation. Y.L. Zhang proposed channel attention (CA) mechanism to adaptively rescale channel-wise features by considering interdependencies among channels. To consider the channel attention and space attention jointly, Y.T. Hu introduced the channel and spatial attention block (CBAM) [14] module into deep SR network [9], where a set of channel-wise and spatial attention residual (CSAR) blocks was conducted and stacked in a chain structure to dynamically modulate multi-level features in a global-and-local manner. Lately, more attention mechanisms has been applied in super resolution [15,16]. Tao Dai presented a second-order attention network (SAN [15]) that employs repeated local-source residual attention groups (LSRAG) to learn increasingly abstract feature representations. In SAN, a novel trainable second-order channel attention (SOCA) module was developed to adaptively rescale the channel-wise features by using second-order feature statistics for more discriminative representations. Further, L.G. Wang created a parallax-attention mechanism (PASSRnet [16]) to integrate the information from a stereo image pair, handling different stereo images with large disparity variations.

Although these existing attention-based approaches have made good efforts to improve SR performance, the reconstruction of rich details for SISR is still a challenge. In deep networks, the low resolution (LR) inputs and extracted features contain different types of information across channels, locations, and layers, which have different reconstruction contributions for reasons. However, the common convolutional layer imposes locality and translation equivariance via a limited receptive and weight sharing, respectively. The local nature of the convolutional kernel prevents it from capturing global contexts in an image, which is necessary for the details of SR images. Consequently, contributions across different aspects are not equal, which causes that multiple feature maps cannot be fully utilized.

**Figure 2:**
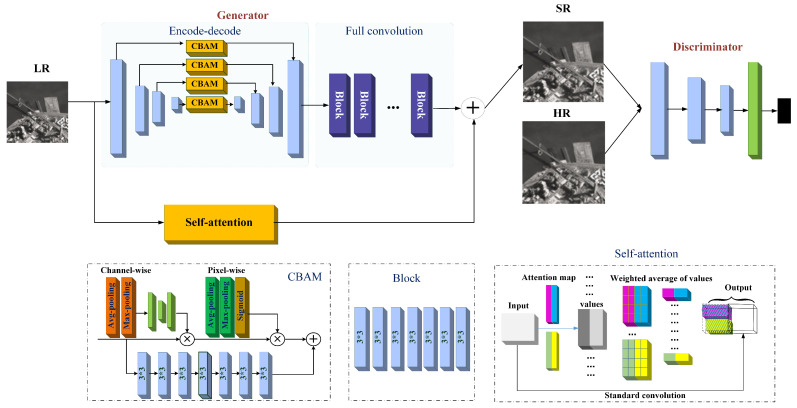
Architecture of self augmented attention (SAA)-WGAN.

Inspired by the above observations, we propose a method that can capture the global contexts by attention-augmented convolution and extract multi-scale features via an encode-decode network. The features from attention-augmented convolution and the encode-decode network are shown in Figure 2. Features of attention-augmented remain lots of details, such as corners and edges. It further assists the HR image reconstruction in the spatial domain and can be concatenated with the multi-scale feature.

## 3. Method

Our system reconstructs high-resolution images via Wasserstein generative adversarial networks with the channel and spatial attention to obtain more representative features. Especially, the attention of channel and spatial is a flexible mechanism to capture information of channel features and position features in a self-adaptive manner such that accumulated important information is weighted highly. Besides, a WGAN network with a comprehensive loss function is applied to achieve a realistic display of SR reconstruction results with more details.

### 3.1. Architecture

The architecture of the self augmented attention WGAN (SAA-WGAN) is illustrated in Figure 2. It consists of two parallel branches, including an encode-decode network and a self attention network. The encode-decode network is composed of two modules, i.e., the encode-decode module (EDM) and the fully convolutional network (FCN). The FCN involves five convolution blocks of eight kernels with a size of 3 × 3. The EDM is a four-scale encode-decode convolutional module, and the CBAM is rubbed into each scale to enhance multi-scale feature representation. Meanwhile, self augmented attention (SAA) convolution is introduced to make a relation of the space and the channel feature subspace for a powerful convolution.

**Wasserstein GAN**. Wasserstein GAN is proposed by Martin Arjovsky and others to optimize a discriminator by maximizing the Earth Mover (EM) distance between the discriminative result of fake and real samples. Thus, the “discriminator” is not a direct critic of telling the fake samples apart from the real ones anymore. Instead, it is trained to learn a K-Lipschitz continuous function (satisfy ${∥f∥}_{L}⩽K$) to help compute Wasserstein distance [17] which is linear for the entire sample space. The Wasserstein distance is informally defined as

(4) $$\begin{array}{c} \underset{G}{min}\underset{D}{max}-\left(-E\left[D\left(I,x\right)\right]+E\left[D\left(I,\hat{x}\right)\right]\right), \end{array}$$

where *I* is LR image; *x* is the real image; and $\hat{x}$ is SR image. Loss is the set of 1-Lipschitz functions. The discriminator loss is

(5) $$\begin{array}{c} \underset{D}{Loss}=-E\left[D\left(I,x\right)\right]+E\left[D\left(I,\hat{x}\right)\right]. \end{array}$$

In practice, a modified generator loss is expressed as

(6) $$\begin{array}{c} \underset{G}{Loss}=-E\left[D\left(I,\hat{x}\right)\right]. \end{array}$$

Wasserstein GAN removes the logarithm for continuous gradient update and uses gradient penalty for the relevance of parameters and constraints. It solves some problems, such as the unstable gradient of the generator and insufficient diversity of generated data, in GAN. So, it is used in our model to facilitate the reconstruction of more detail in SR image.

**Figure 3:**
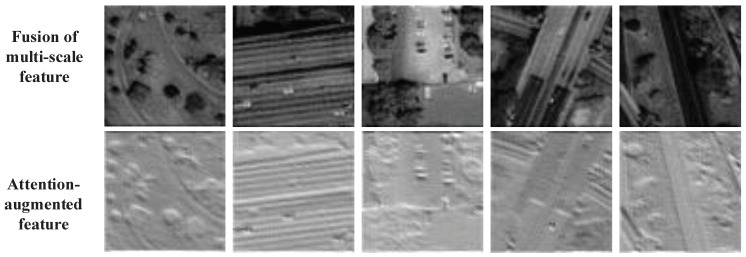
Attention-augmented results.

**Channel and spatial attention block (CBAM).** Attention model has been used to help the network to focus on the features which are more critical for the performance. In our model, to fully exploit its information, we utilize a channel and spatial attention block for the feature re-enhancement. The CBAM adopts average-pooling to squeeze the spatial dimension of the input feature map to achieve channel attention. It also applies average-pooling and max-pooling operations along channel axis for spatial attention, then concatenates these features, and generates a spatial attention map by a convolution layer. In this way, the weights of different channels and different positions can be flexibly adjusted under the importance of the information. The input of CBAM is the feature of each layer in EDM, and the multi-scale features captured by CBAM are displayed in the first row of Figure 3.

**Figure 4:**
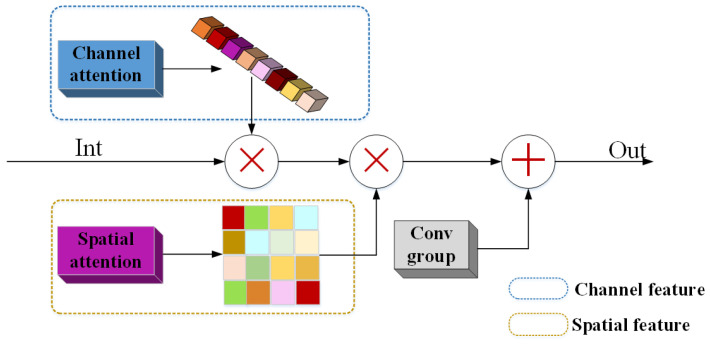
Channel and spatial attention block (CBAM).

The CBAM structure as Figure 4, CBAM feature math expression can be inferred by a 1D channel attention map $M_{c}$ and a 2D spatial attention map $M_{s}$, and the CBAM feature $F_{c}bam$ can be expressed as

(7) $$\begin{array}{c} F_{c}bam=M_{s}\left(F\right)⨂M_{c}\left(F\right)⨂F, \end{array}$$

(8) $$\begin{array}{c} M_{c}\left(F\right)=\sigma \left(W_{1}\left(AvgPool\left(F\right)\right)+W_{1}\left(MaxPool\left(F\right)\right)\right)=\sigma (W_{1}\left(W_{0}\left(F_{avg}^{c}\right)\right)+W_{1}\left(W_{0}\left(F_{max}^{c}\right)\right), \end{array}$$

(9) $$\begin{array}{c} M_{s}\left(F\right)=\sigma \left(f^{7\times 7}\left(\left[AvgPool\left(F\right)\right);\left(MaxPool\left(F\right)\right]\right)\right)=\sigma \left(f^{7\times 7}\left(\left[F_{avg}^{s},F_{max}^{s}\right]\right)\right), \end{array}$$

where ⨂ denotes element-wise multiplication. AvgPool() is average-pooling, and MaxPool() is max-pooling. $F_{avg}^{c}$ and $F_{max}^{c}$ denote channel average-pooled features and max-pooled channel features, respectively. $F_{avg}^{s}$ and $F_{max}^{s}$ denote average-pooled spatial features and max-pooled spatial features, respectively. σ denotes the sigmoid function, and $f^{7\times 7}$ represents a convolution operation with the convolutional filter size of 7×7. $W_{1}$ and $W_{0}$ are feature weights after pooling and after activation.

**Self augmented attention (SAA) convolution**. Self augmented attention (SAA) convolution aims to compute a weighted average of values from hidden units, and the weights are produced dynamically via a similarity function. It can also capture long-range interactions among input signals and gives the dynamical weights obtained by hidden units to the input. The SAA takes local information and re-calibrated global information into the convolution. That is, two heads of feature subspace can participate in the attention mechanism to get both spatial and channel-wise weighted maps, which are used to re-weight the corresponding location of the input image, and, finally, concatenates with the point-wise convolution to achieve the enhanced convolution operation in SAA. Therefore, augment convolutions are applied to the self-attention mechanism for representative and abstract features, as displayed in the second row of Figure 3.

**Figure 5:**
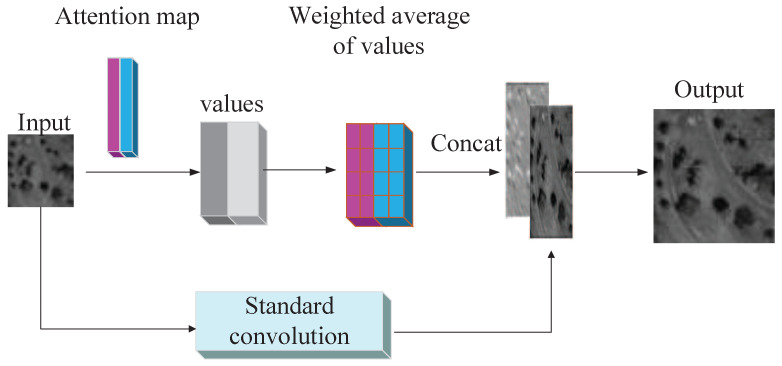
Attention-augmented convolution results.

Self attention-augmented convolution is achieved by concatenating convolutional feature maps into self-attentional feature maps which is capable of modeling longer range dependencies (see Figure 5). First, we flatten the input matrix X shape of $\left(H,W,d_{in}\right)$ to $\left(HW,d_{in}\right)$ and take an operation of multi-head attention as the transformer architecture [8]. The output of the self-attention module for a head *h* can be formulated as:

(10) $$\begin{array}{c} O_{h}\left(X\right)=Softmax\left(\frac{\left(XW_{q}\right){\left(XW_{k}\right)}^{T}}{\sqrt{d_{k}^{h}}}\right)\left(XW_{v}\right), \end{array}$$

where $W_{q}$, $W_{k}$∈$R^{d_{in}\times d_{k}^{h}}$, and $W_{v}$∈$R^{d_{in}\times d_{v}^{h}}$ are learned linear transformations used to map the input *X* to queries $Q=XW_{q}$, keys $K=XW_{k}$, and values $V=XW_{v}$. Attention (Q,K,V) map uses query *Q* and keys *K* matrix as weight of values *V*, and we can obtain a HW×HW matrix via $\left(XW_{q}\right){\left(XW_{k}\right)}^{T}$. Then, the outputs of all heads (1, 2, 3, …, *h*) are then concatenated as follows:

(11) $$\begin{array}{c} O_{atten}\left(X\right)=Concat\left[O_{1},O_{2},O_{3},\ldots ,O_{h}\right]W^{o}, \end{array}$$

where $W^{o}\in R^{dv\times dv}$ is a linear transformation. $O_{atten}\left(X\right)$ is then reshaped into a tensor of shape (H,W,dv) to match the original spatial dimensions.

The comprehensive feature $F_{sr}$ is expressed by SAA feature $F_{self}$ and FCN feature $F_{fcn}$.

(12) $$\begin{array}{c} F_{sr}\left(\cdot \right)=C\left(F_{self},F_{fcn}\right), \end{array}$$

(13) $$\begin{array}{c} F_{cn}=F_{c}\left(\sum Fi_{cbam}\right), \end{array}$$

where C(·) function is sum of $F_{fcn}$ and $F_{self}$, and $F_{c}$ function is series of convolution in FCN module. $\sum Fi_{cbam}$ is the fusion $Fi_{cbam}$(*i* = 1, 2, 3, 4), $Fi_{cbam}$ is the CBAM feature, $F_{self}$ is the SAA feature, and $F_{fcn}$ is the FCN feature.

### 3.2. Loss function

Efficient loss functions and deep CNN networks have been exploited in other SR methods [18]. To achieve better performance, we utilize the pixel-wise loss (e.g., L1 loss) to minimize the error between the real image and SR result in pixel-level, which has been widely used in many image reconstruction problems [19]. The pixel-wise loss can get excellent performance in Peak Signal to Noise Ratio (PSNR) but always introduces some artifacts. To avoid image artifacts, we also introduce the perceptual loss in our model. Perceptual loss tries to reduce the feature gap between the real image and the reconstructed image at certain layers of VGG19 features, and it can be used to preserve semantic information and achieve better visual quality. In addition, the gradient loss is used to minimize the gradient difference between the real image and the reconstructed image in different directions. The combination of pixel-wise loss, gradient loss, and perceptual loss is applied to supervise the training process. The combined generator loss can be expressed as

(14) $$\begin{array}{c} \underset{G}{Loss_{final}}\left(I,\hat{x}\right)=-\left[a*loss_{pix}\left(I,\hat{x}\right)+b*loss_{per}\left(I,\hat{x}\right)+c*loss_{grad}\left(I,\hat{x}\right)\right]. \end{array}$$

In addition, the discriminator loss is

(15) $$\begin{array}{c} \begin{array}{c} \underset{D}{Loss_{final}}\left(x,\hat{x}\right)=\left[a*loss_{pix}\left(I,\hat{x}\right)+b*loss_{per}\left(I,\hat{x}\right)+c*loss_{grad}\left(I,\hat{x}\right)\right]\\ -[a*loss_{pix}\left(I,x\right)+b*loss_{per}\left(I,x\right)+c*loss_{grad}\left(I,x\right)] \end{array}. \end{array}$$

Here, $loss_{pix}$, $loss_{per}$ and $loss_{grad}$ denote the pixel-wise loss, perceptual loss, and gradient loss, respectively, $loss_{pix}={∥I-\hat{x}∥}_{2}$, $loss_{grad}={∥\nabla \left(I\right)\nabla \left(\hat{x}\right)∥}_{2}$, $loss_{per}={∥\delta_{l}\left(I\right)-\delta_{l}\left(\hat{x}\right)∥}_{1}$; *a*, *b*, and *c* are weighted values which are adjusted according to the training situation. ∇() is gradient computation, $\delta_{l}\left(\right)$ is the feature map of the *l*th layer of VGG19 (*l* = 1, 2, 3, 4, 5), ${\left\|\right\|}_{1}$ indicates L1 norm, and ${\left\|\right\|}_{2}$ indicates L2 norm.

We conducted the three experiments using different combination of these losses to validate the effectiveness of the comprehensive loss. It can be seen from Table 1 that the loss acted on the generative network can improve the performance as expected, from 32.23 dB to 33.29 dB. These comparisons firmly demonstrate the effectiveness of loss.

## 4. Experimental Evaluation

In this part, we conduct experimental comparison of state-of-art deep learning methods, including SRCNN [20], VDSR [21], EDSR [22], LapSRN [23], RCAN [7], ESPCN [24], RDN [25], SRGAN [11], and CGAN [26]. And the baselines are re-implemented based on the source-code that the authors provided. We implement our models with the TensorFlow framework and train them using NVIDIA Titan V GPU. In the following subsection, we will provide reasonable settings for the implementation details and parameters in our SR model.

### 4.1. Implementation Details

We use the DIV2K dataset, a high-quality (2K resolution) dataset with 800 images, for our training. The training samples are randomly cropped from the original images with a fixed size of 64 × 64.

The generative model is trained using the loss function in Equation (11) with a = 0.22, b = 0.43, c = 0.35. The learning rate is initialized as $1\times 10^{-4}$ and decayed by a factor of 1 every $1\times 10^{5}$ of mini-batch updates. For optimization, we used Adam with $\beta_{1}$ = 0.9, $\beta_{2}$ = 0.999, $\varepsilon =e^{-10}$, and step size α=0.001. We alternately updated the generator and discriminator network until the model converges.

### 4.2. Comparisons and Results

To validate the effects of self-attention, we carried out a series of experiments involving the following three parts:

- Comparison test between castrated model without SAA convolution and SAA-WGAN.
- Comparison test on PAN datasets of DOTA and GEO, evaluation index of PSNR and SSIM on GEO images.
- Comparison with benchmark networks on classic datasets, including Set5, Set14, BSD100, and Urban100.

**Figure 6:**
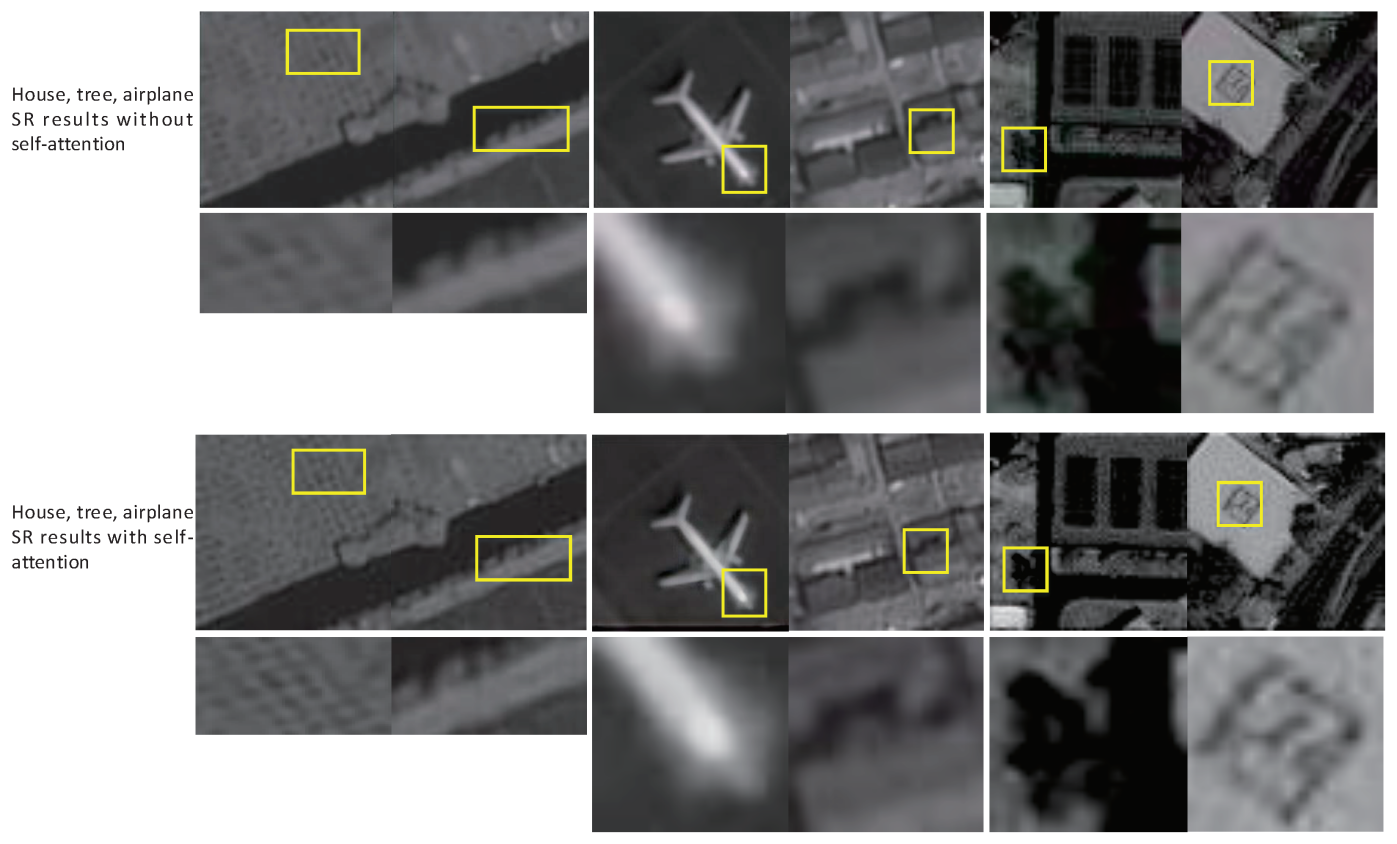
Panchromatic (PAN) image super-resolution (SR) from GEO.

First, we test the performance of SAA-WGAN and the castrated model without SAA on the DOTA dataset, and some results are shown in Figure 6. The details of the airplane, the shade of the tree, and the house in Figure 6 have been significantly improved because of SAA ability to keep long-distance details, indicating that self-attention could improve the network performance.

**Figure 7:**
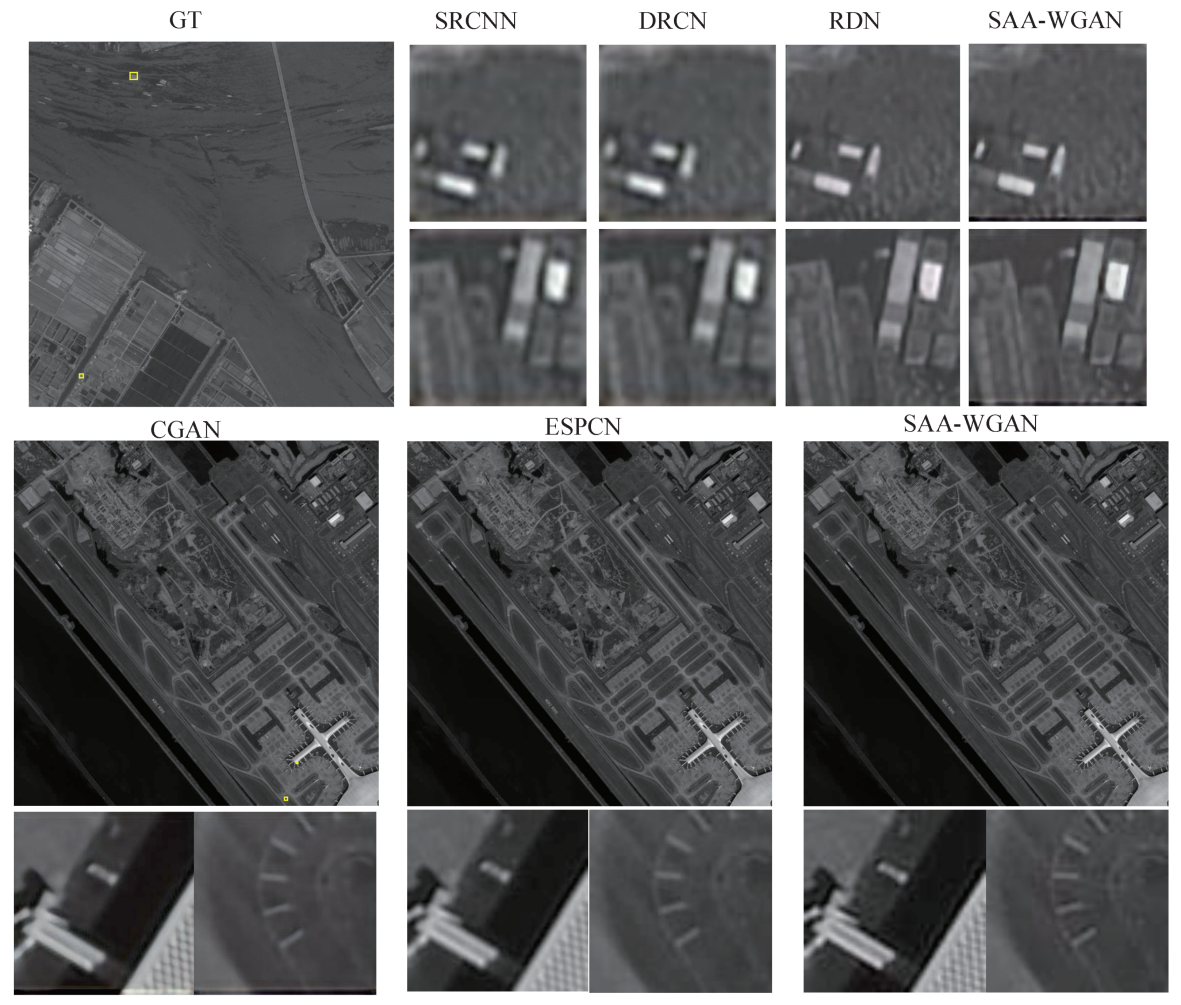
DOTA image for x4 scale SR.

**Figure 8:**
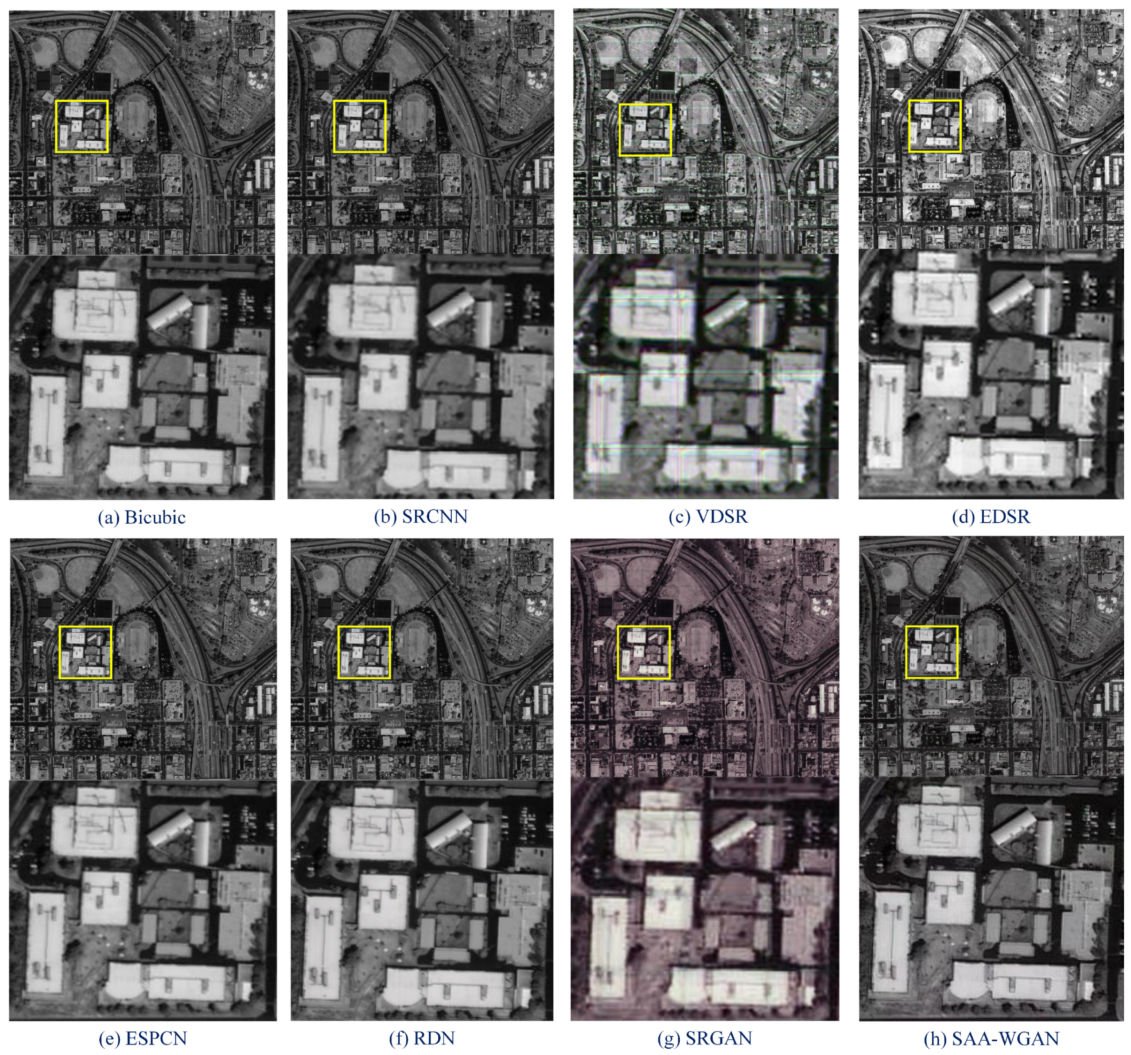
PAN image SR from GEO (scale = 4).

**Figure 9:**
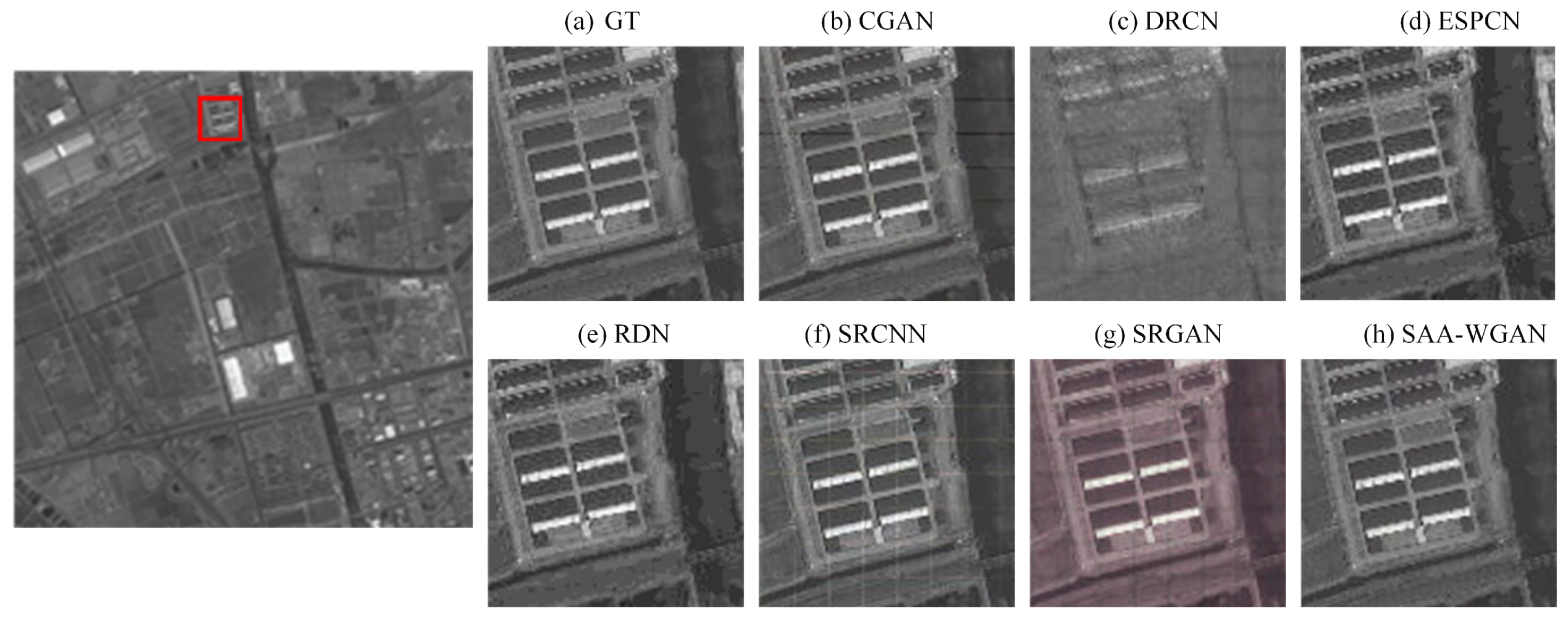
SR results from DOTA datasets (scale = 4).

Then, comparisons are conducted on DOTA and GEO images using state-of-art algorithms, which proves the performance superiority of SAA-WGAN. The results of the SAA-WGAN are displayed in Figure 7, Figure 8 and Figure 9. We show visual comparisons of different benchmark algorithms on scale ×4 in Figure 7. As can be seen, all the compared methods suffer from blurring artifacts with varying degrees, failing to recover more details. However, our SAA-WGAN can recover them obviously, showing more faithful to the ground truth. Due to the resolution of the image is too high, the size of PAN images are cropped into 64 × 64.

To further illustrate the universality advantage on other datasets of SAA-WGAN, we compare our method with 8 state-of-the-art methods (Bicubic, SRCNN, SCN, VDSR, LapSRN, EDSR, RDN, RCAN) on some most used SR dataset, e.g., Set5, Set14, BSD100, Urban100. More comparisons about PSNR/SSIM are provided in Table 2. It shows quantitative comparisons for ×2, ×4, and ×8 SR. The best results are annotated with blue text in Table 2. It demonstrates that our method almost achieves the best performance on all the datasets with all scaling factors.

We also find that, when the scaling factor becomes larger (e.g., 8), the PSNR gain of our method also becomes larger. When the scale factor is 2, the PSNR gain of our method tested on BSD100 and Urban100 exceeds RCAN by 1.5 dB and 1.2 dB, respectively. Similarly, on the same two datasets with the scale factor of 4, the proposed method has more gains than RCAN of 2.2 dB and 1.4 dB, respectively. When the scale factor is 8, the PSNR gain of this method exceeds RCAN by 2.99 dB and 1.97 dB, respectively. This observation shows that deeper network structure and powerful attention mechanism can improve network performance.

**Figure 10:**
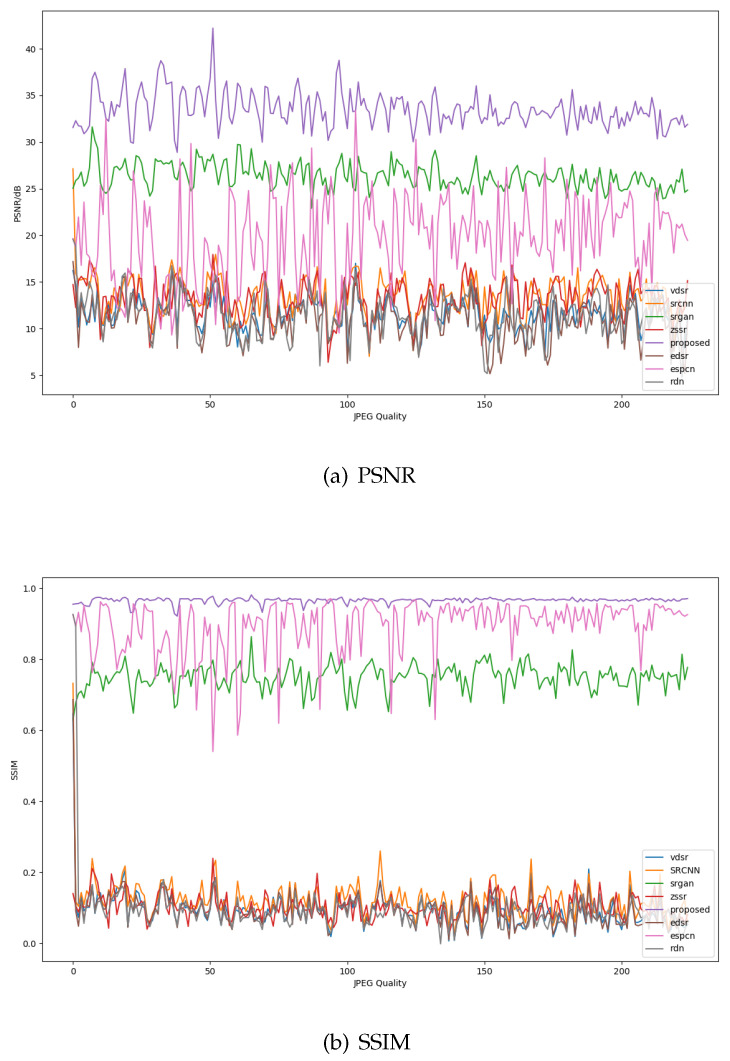
The metrics of GEO image (The proposed SAA-WGAN is the purple curve.).

Figure 10 is the objective evaluation on image patches of Figure 8. In comparison algorithms, the performance curves of the SRGAN and ESPCN are significantly higher than the other five algorithms. Although SRGAN uses discriminative network that can extract the semantic information to get more useful features, ESPCN adopts a reconstruction strategy of concentrating multiple channel features to form a fused feature map which uses the relationship across channels. However, the evaluation indicators of SRGAN and ESPCN cannot exceed the proposed method. The PSNR of SAA-WGAN reaches 32 dB, and the SSIM curve fluctuates around 0.92; it is achieved by its ability of extracting attention feature from hidden units using SAA and CBAM, which is superior to other comparison algorithms in subjective vision and objective evaluation.

## 5. Conclusions

We propose a self attention-augmented network SAA-WGAN for PAN image SR. SAA-WGAN uses the EDM to extract multi-scale information and utilizes FCN to reconstruct HR images. The CBAM and the SAA are rubbed into the SAA-WGAN to enhance multi-scale feature representation and make use of the relationship in both spatial and channel subspaces. Further, the pixel loss, perceptual loss and gradient loss are combined to supervise the training process. Extensive experiments on benchmark datasets and PAN images demonstrate the effectiveness of our proposed SAA-WGAN.

## Figures and Tables

**Table 1:** Test of different loss on the Set5 dataset.

Loss	${\mathrm{loss}}_{\mathrm{pix}}$	${\mathrm{loss}}_{\mathrm{per}}$	${\mathrm{loss}}_{\mathrm{grad}}$	PSNR
1	✓	✕	✕	32.23
2	✕	✓	✓	32.37
3	✓	✓	✕	32.45
4	✓	✕	✓	32.71
5	✓	✓	✓	**33.29**

**Table 2:** SR results of benchmark.

Image Index	Scale	Set5		Set14		BSD100		Urban100	
		PSNR	SSIM	PSNR	SSIM	PSNR	SSIM	PSNR	SSIM
Bicubic	×2	33.66	0.9299	30.24	0.8688	29.56	0.8431	26.88	0.8403
SRCNN	×2	36.66	0.9542	32.45	0.9067	31.36	0.8879	29.50	0.8946
SCN	×2	37.05	0.9576	33.17	0.9120	31.56	0.8923	30.32	0.9021
RDN	×2	38.24	0.9614	34.01	0.9212	32.34	0.9017	32.89	0 9353
VDSR	×2	37.53	0.9590	33.05	0.9130	31.90	0.8960	30.77	0.9140
EDSR	×2	38.11	0.9602	33.92	0.9195	32.32	0.9013	32.93	0.9351
LapSRN	×2	37.52	0.9591	33.08	0.9130	31.05	0.8950	30.41	0.9101
RCAN	×2	38.27	0.9614	34.12	0.9216	32.41	0.9027	33.34	0.9384
**SAA-WGAN**	×2	38.34	0.9733	34.71	0.9310	33.91	0.9130	34.53	0.9453
Bicubic	×4	28.42	0.8104	26.00	0.7027	25.96	0.6675	23.14	0.6577
SRCNN	×4	30.48	0.8628	27.50	0.7513	26.90	0.7101	24.52	0.7221
DRCN	×4	31.45	0.8714	28.00	0.7677	27.14	0.7312	25.67	0.7556
RDN	×4	32.47	0.8990	28.81	0.7871	27.72	0.7419	26.61	0.8028
VDSR	×4	31.35	0.8830	28.02	0.7680	27.29	0.7260	25.18	0.7540
EDSR	×4	32.46	0.8968	28.80	0.7876	27.71	0.7420	26.64	0.8033
LapSRN	×4	31.45	0.8850	28.19	0.7720	27.32	0.7270	25.21	0.7560
RCAN	×4	32.73	0.9013	28.98	0.7910	27.85	0.7455	27.10	0.8142
**SAA-WGAN**	×4	33.03	0.9115	29.45	0.8110	29.65	0.9101	28.53	0.8372
Bicubic	×8	24.40	0.6580	23.10	0.5660	23.67	0.5480	20.74	0.5167
SRCNN	×8	25.33	0.6900	24.13	0.5660	21.29	0.5440	22.46	0.6950
SCN	×8	25.59	0.7071	24.02	0.6028	24.30	0.5698	21.52	0.5571
VDSR	×8	25.93	0.7240	24.26	0.6140	24.49	0.5830	21.70	0.5710
EDSR	×8	26.96	0.7762	24.91	0.6420	24.81	0.5985	22.51	0.6221
LapSRN	×8	26.15	0.7380	24.35	0.6200	24.54	0.5860	21.81	0.5810
DRRN	×8	24.87	0.8290	24.81	0.7734	20.79	0.7968	21.84	0.7896
RCAN	×8	27.31	0.7878	25.23	0.6511	24.98	0.6058	23.00	0.6452
**SAA-WGAN**	×8	26.17	0.8338	25.33	0.7742	27.97	0.8816	24.97	0.8224

## Data Availability

DOTA dataset is available at https://captain-whu.github.io/DOTA.
